# Detection of small ruminant Lentivirus proviral DNA in red deer from Poland

**DOI:** 10.1186/s12917-024-04059-y

**Published:** 2024-05-13

**Authors:** Monika Olech, Marta Parzeniecka-Jaworska

**Affiliations:** 1 Department of Pathology, National Veterinary Research Institute, Pulawy, 24-100 Poland; 2 Department of Small Animal Diseases and Clinic, Faculty of Veterinary Medicine, Warsaw University of Life Sciences, Warsaw, 02-766 Poland

**Keywords:** SRLV, CAEV, Red deer, Wild ruminants, Cross-species transmission, Phylogenetic analysis, Nested PCR

## Abstract

Small ruminant lentiviruses (SRLVs) are widespread and infect goats and sheep. Several reports also suggest that SRLVs can infect wild ruminants. The presence of specific antibodies against SRLVs has been identified in wild ruminants from Poland, but no studies have been conducted to detect proviral DNA of SRLVs in these animals. Therefore, the purpose of this study was to examine samples from Polish wild ruminants to determine whether these animals can serve as reservoirs of SRLVs under natural conditions. A total of 314 samples were tested from red deer (*n* = 255), roe deer (*n* = 52) and fallow deer (*n* = 7) using nested real-time PCR. DNA from positive real-time PCR samples was subsequently used to amplify a CA fragment (625 bp) of the *gag* gene, a 1.2 kb fragment of the *pol* gene and an *LTR-gag* fragment. Three samples (0.95%) were positive according to nested real-time PCR using primers and probe specific for CAEV (SRLV group B). All the samples were negative for the primers and probe specific for MVV (SRLV A group). Only SRLV *LTR-gag* sequences were obtained from two red deer. Phylogenetic analysis revealed that these sequences were more closely related to CAEV than to MVV. Our results revealed that deer can carry SRLV proviral sequences and therefore may play a role in the epidemiology of SRLVs. To our knowledge, this is the first study describing SRLV sequences from red deer.

**Supplementary Information:**

The online version contains supplementary material available at 10.1186/s12917-024-04059-y.

## Introduction

Visna-maedi virus (MVV) and Caprine arthritis encephalitis virus (CAEV), which belong to the *Lentivirus* genus in the *Retroviridae* family, are members of the small ruminant lentivirus (SRLV) group that infect sheep (family *Bovidae*, subfamily *Caprinae*, genus *Ovis*) and goats (family *Bovidae*, subfamily *Caprinae*, genus *Capra*). MVV and CAEV were originally considered species-specific pathogens for sheep and goats, respectively, but many research groups have reported that these viruses cross the species barrier and infect both species [1–3]. Therefore, a new classification system for SRLVs has been proposed in which there are five main (A-E) genetic groups, which are further divided into several subtypes. Group A corresponds to heterogeneous MVV-like viruses, while group B refers to genetically less complex CAEV-like viruses. Other groups (C, D and E) were identified on the basis of their high genetic divergence from the two previous groups. However, the group D strains appeared to be group A strains, showing a variation in the gene *pol* [3–5].

SRLVs are small enveloped RNA viruses that cause persistent infection and slow disease progression in their hosts, despite specific antiviral immune responses. After infection, SRLV integrates into host cell DNA as a provirus, with tropism mainly for monocytes, macrophages, and dendritic cells. The proviral DNA contains structural (*gag*, *pol* and *env*) and regulatory (*vif*, *vpr-like*, and *rev*) genes flanked by noncoding long terminal repeat regions (LTRs). SRLVs mainly affect organs such as the lungs, joints, mammary gland and central nervous system, but these viruses have also been found in the liver, spleen, kidney, lymph node, thyroid follicles and intestinal enterocytes [6, 7]. SRLV infections persist throughout life and are characterized by four main disease syndromes—arthritis, mastitis, interstitial pneumonia and encephalomyelitis; however, most infected animals never develop clinical signs. There is no successful vaccine or treatment for SRLVs, so these viruses are widespread among sheep and goats worldwide. The most likely routes of SRLVs transmission are ingestion of SRLV-contaminated milk or colostrum or via inhalation of respiratory secretions through close contact between animals. However, transmission through contaminated feeding and drinking equipment, contaminated water and feces is also possible [4, 8–10].

Although cross-species transmission of SRLVs between sheep and goats has become evident, research on SRLV infections in wild ruminants is very limited. Antibodies against SRLVs have been detected in Rocky Mountain goats in the USA [11], red deer and roe deer in Spain, and mouflons in Slovenia and Spain, but the number of positive samples was very low (single positive results) [12, 13]. No reaction to SRLV infection was found in wild mouflons in Spain [14], red deer in California [15, 16], chamois in Italy [17] or Slovenia [12] or in several species (344 from the sera of *Capreolus capreolus*, *Cervus elaphus* and *Dama dama*) of wild ruminants in Germany [18]. To date, natural infection with SRLVs has been confirmed in Rocky Mountain goats (*Oreamnos americanus*) and Alpine ibexes (*Capra ibex*), while experimental infection with SRLVs has been reported in mouflons (*Ovis aries musimon*), which may suggest that wild ruminants may serve as reservoirs of SRLVs [11, 19–21].

Recent serological studies performed in Poland revealed that the prevalence of SRLVs in wild ruminants ranged from 2.5 to 24.6%, depending on the antigen used in the ELISA test [22]. Although specific antibodies against SRLVs have been identified, direct evidence for the presence of the virus in wild small ruminants from Poland is lacking. Therefore, the purpose of this study was to examine samples from Polish wild ruminants (red deer, roe deer and fallow deer) to determine whether these animals can serve as reservoirs of SRLVs under natural conditions.

## Materials and methods

### Animal samples

**Fig. 1:**
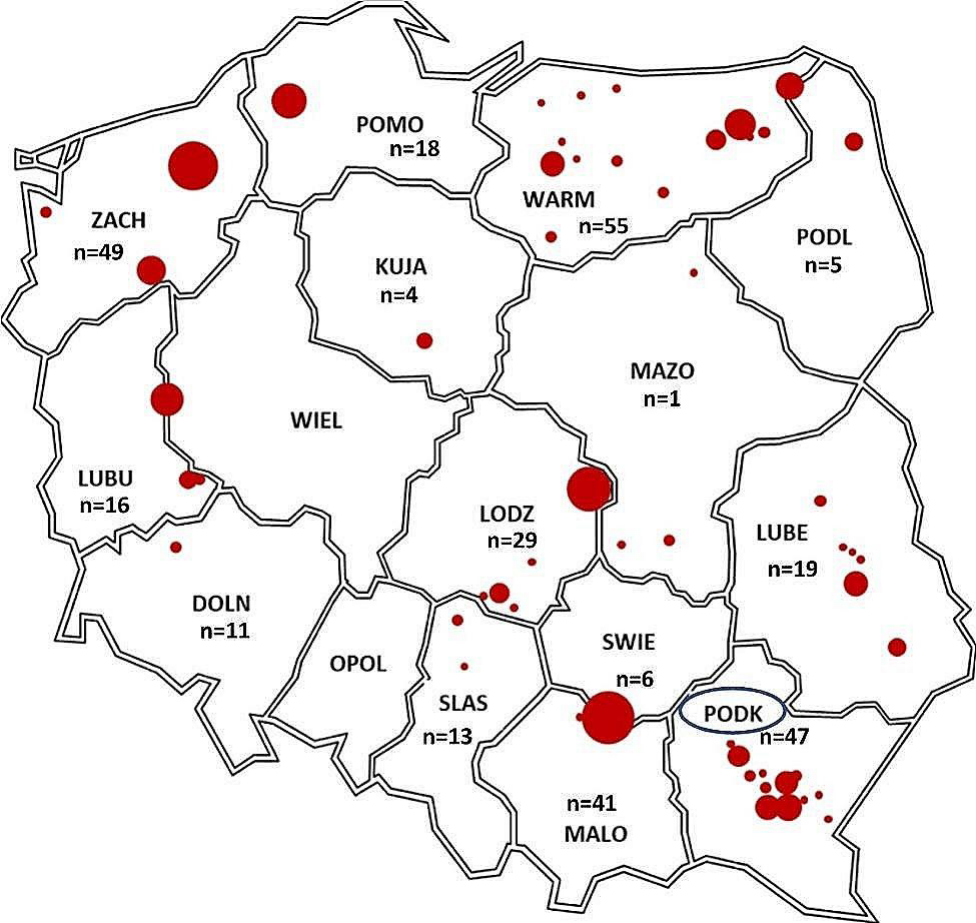
Geographic distribution of samples collected from wild ruminants in Poland in this study. Dots show the areas of sample collection. n-the number of samples collected from the particular voivodship. The voivodeship where the positive samples were detected was marked in a circle

The samples used in this study were obtained from 314 wild ruminants. All the samples were collected from cervids (family of *Cervidae*), including red deer (*n* = 255), roe deer (*n* = 52) and fallow deer (*n* = 7). The samples were collected during hunting seasons (2010–2014 and 2022/2023) and originated from 14 out of 16 voivodships (Fig. 1). DNA was extracted from whole blood collected as blood clots from the jugular vein or heart or from selected organs such as the lung, liver or spleen. No ethical approval was needed, as all samples were collected postmortem during evisceration by the licensed hunters. All methods were carried out in accordance with relevant guidelines and regulations. DNA extraction was performed using the Qiagen DNeasy® Blood & Tissue Kit (Qiagen, Hilden, Germany) according to the manufacturer’s protocol. The DNA concentration and the 260/280 nm ratio were measured spectrophotometrically using a NanoDrop 2000 (Thermo Fischer Scientific, Wilmington, Delaware, USA), and the DNA was stored at -20 °C until use.

### Nested real-time PCR assay

Nested real-time PCR was performed to amplify the LTR-gag region as previously described by Olech et al. [23]. The first pair of primers amplified the target fragment via conventional PCR with a final volume of 25 µL of reaction mixture containing 2 U of OptiTaq DNA polymerase (EURx, Gdansk, Poland), 1xPCR buffer, 1.5 mM MgCl2, 300 nM of each primer, 0.2 mM dNTPs and 1 µg of extracted DNA. The second round of PCR was performed and monitored via real-time PCR. The reaction was performed using a QuantiTect Probe PCR Kit (Qiagen, Hilden, Germany) with a reaction mixture containing 10 µl of 2x QuantiTect Probe PCR Master Mix, 900 nM each primer, 200 nM each specific probe and 5 µl of the PCR product from the first amplification. In the second reaction, all the samples were tested separately with primers and probes specific for MVV and CAEV-like viruses. To establish a standard for these assays, the target *LTR-gag* regions of the SRLV A5 and B1 strains were amplified and cloned. The reference plasmid was subsequently used to generate a standard curve based on 10-fold serial dilutions (from 10^9^ to 10^1^).

### Conventional PCR, sequencing and analysis

Polymerase chain reaction (PCR) was performed using genomic DNA isolated from 3 red deer that were positive according to nested real-time PCR. The CA (625 bp) fragment of the *gag* gene, the 1.2 kb fragment of the *pol* gene and the *LTR-gag* fragment were amplified by nested PCR. PCRs were performed as previously described [5, 24, 25]. Positive PCR products were purified using NucleoSpin Gel and PCR Clean-up (Marcherey-Nagel, GmbH 7 Co, Hamburg, Germany) and inserted into the pDRIVE vector (Qiagen, GmbH, Hilden, Germany). Plasmid DNA was transformed into competent cells and extracted using a NucleoSpin Plasmid Kit (Marcherey-Nagel, GmbH 7 Co., Hamburg, Germany). Positive clones were sequenced by a private company (Genomed S.A., Warsaw, Poland). The obtained SRLV sequences were trimmed and analyzed using Geneious Pro 5.3 software (Biomatters Ltd., Auckland, New Zealand). Then, the consensus sequences were aligned with other sequences retrieved from GenBank using the clustalW algorithm implemented in MEGA 6 software [26]. The phylogenetic tree was inferred using the neighbor joining method and the Kimura 2-parameter model with the gamma distribution with the five rate categories (K2 + G). Pairwise genetic distance was estimated using the p-distance substitution model. The robustness of the nodes was evaluated by nonparametric bootstrap analysis with 1000 replicates. The percent nucleotide acid sequence identity (percentage of identical bases/residues) was estimated using Geneious software. All novel sequences reported in this study were deposited in the GenBank database under accession numbers PP054400-PP054401.

## Results

DNA samples originating from 314 wild ruminants were tested via nested real-time PCR using primers and probe specific for MVV (MVV assay) and primers and probe specific for CAEV (CAEV assay). Only three samples (0.95%) were positive using primers and probe specific for CAEV (sample #2256, #2385 and #2161). All 314 samples were negative when tested using primers and probe specific for MVV. In 311 samples, neither MVV nor CAEV was detected. The positive samples originated from red deer only. Samples from roe deer and fallow deer were negative.

**Fig. 2:**
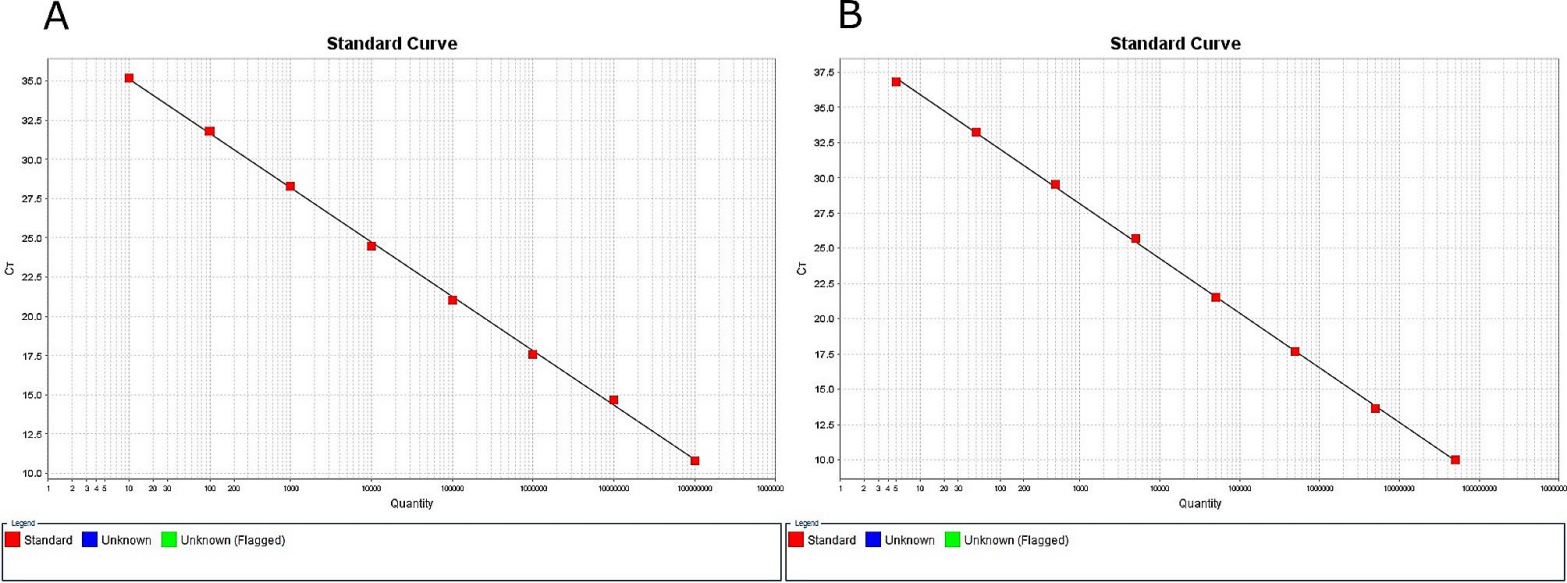
Quantification curves constructed from serial dilution analysis using a recombinant plasmid containing the *LTR-gag* region of SRLV; (**A**) MVV assay; (**B**) CAEV assay; Ct: cycle threshold

The reaction efficiency of the MVV assay ranged from 92 to 100%, and the R^2^ was 0.961–0.999. The CAEV assay showed a reaction efficiency of 84-97.6% and an R^2^ of 0.975–0.997. The limit of detection (LOD) for both assays was 5 genome copies per reaction (Fig. 2). The negative controls produced no detectable fluorescence signals.

**Fig. 3:**
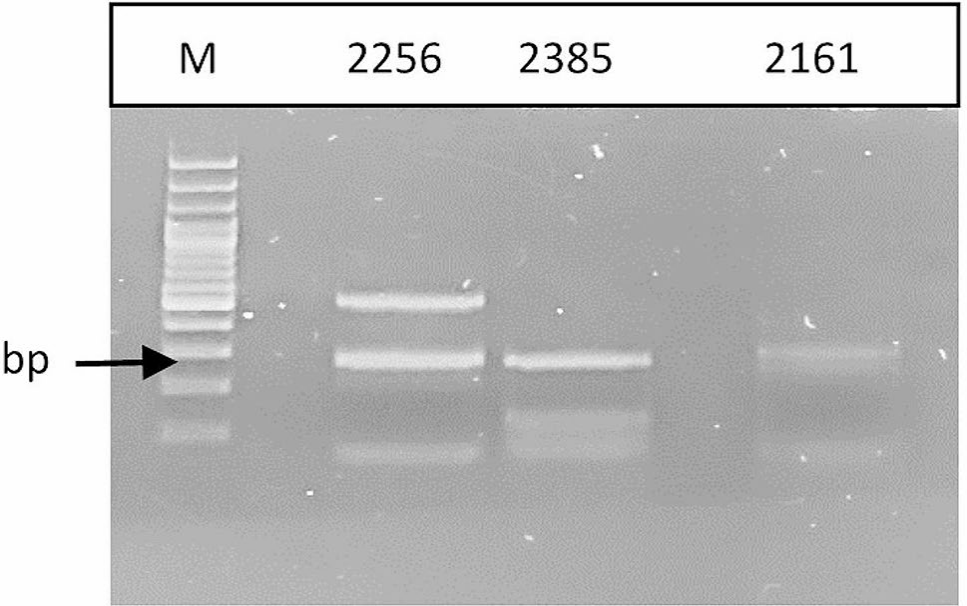
Electrophoretic analysis of *LTR-gag* amplification products. Full-length gels are presented in Supplementary Fig. 1

DNA extracted from positive samples was used as a template for nested PCR to detect the CA fragment of the *gag* gene, the 1.2 kb fragment of the *pol* gene and the *LTR-gag* fragment. All the samples yielded a positive PCR product when the *LTR-gag* fragment was amplified (Fig. 3). No successful amplification was obtained when primers specific for SRLV *gag* or *pol* fragments were used.

**Fig. 4:**
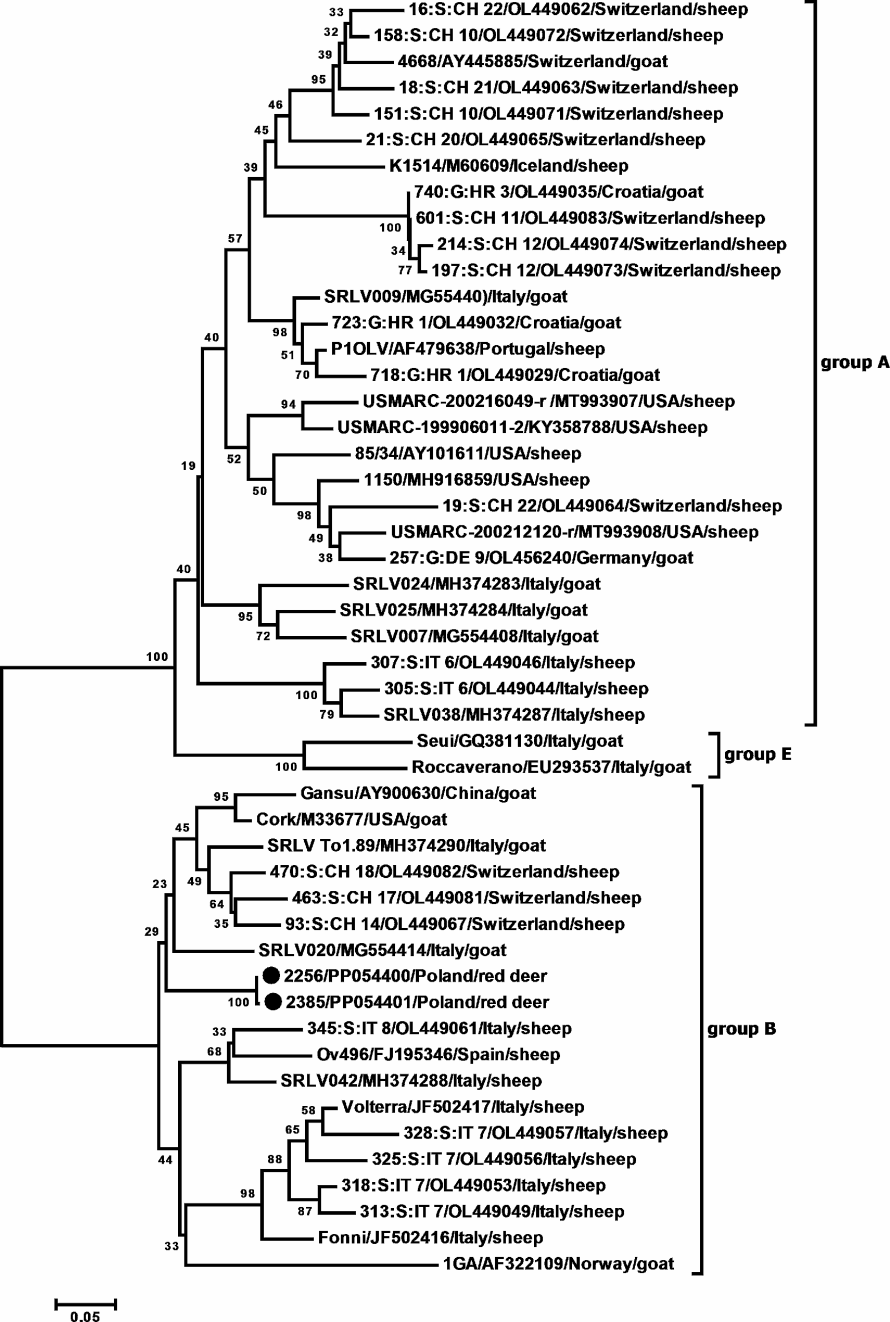
A neighbor-joining phylogenetic tree was constructed using the *LTR-gag* region. Newly characterized sequences are labeled with black circles. Reference sequences are indicated by their names, GenBank accession numbers, country of origin and species origin

After purification, *LTR-gag* PCR products were cloned and sequenced. The BLAST program was used to check sequence similarity. SRLV sequences were obtained from samples #2256 and #2385. Sequences obtained from sample #2161 showed no statistically significant similarity in the BLAST search, so these sequences were not analyzed further. The similarity of the consensus sequences obtained from samples #2256 and #2385 was very high (99%). These consensus sequences were subsequently aligned with 47 sequences representing different SRLV genotypes and subtypes. Phylogenetic analysis clearly showed that the SRLV sequences obtained from red deer were more closely related to CAEV than to MVV (Fig. 4). The mean genetic distance between the SRLV sequences obtained from red deer and the reference strain Cork was 12.0%, while the mean genetic distance between the SRLV sequences obtained from red deer and the reference strain K1514 was 45.2%. *LTR-gag* sequences from red deer showed the highest similarity to the Italian SRLV020 strain (the mean genetic distance of 10.55%), and to the Swiss 93:S: CH 14 strain (genetic distance of 10.5%).

## Discussion

SRLV infection in goats and sheep occurs worldwide, especially in European and North American countries [9]. Consequently, the World Organization for Animal Health (Office International des Epizooties, OIE) has classified SRLVs as the pathogens causing the major infectious disease of small ruminants. In Poland, the overall prevalence of SRLVs in sheep reached more than 9%, while in goats, it was 42% [27, 28]. Molecular studies have shown that the SRLVs circulating in Poland are very heterogeneous, and 12 subtypes have been detected so far (A1, A5, A12, A13, A16, A17, A18, A23, A24, A27, B1 and B2). In addition, interspecies SRLVs transmission between sheep and goats, double infections and SRLV recombination have been confirmed [29].

The transmission of infectious agents from domesticated species to wild ungulates (spillover) has been widely reported, indicating that wildlife animals have been implicated in the epidemiology of many persistent and emerging diseases [30–35]. However, studies on the occurrence of SRLVs in free-ranging animals are limited and are mainly based on the detection of antibodies against SRLVs. In most of these studies, antibodies against SRLVs were detected sporadically or not at all [11–18]. The reason for this may be that commercial diagnostic kits designed for domestic animals were used, although these kits were not approved for wild animals. When in-house ELISAs were used, increased reactivity of the wildlife serum was observed. For example, using modified in-house ELISAs based on synthetic peptides, Sanjose et al. [13] detected SRLV antibodies in 14 of 193 (7%) red deer and 1 of 10 (10%) mouflons; however, when the same samples were tested using a commercial test with protein G, none of the animals were seropositive. A study in Poland showed that the prevalence of SRLVs in wild ruminants ranged from 5.3 to 24.6% with in-house ELISAs, while the estimated prevalence using a modified commercial ELISA was 2% [22]. The lack of antibody detection in Rocky Mountain goats harboring SRLVs proviral DNA indicates poor cross-reactivity with the SRLV antigens used in commercial ELISA [19]. Therefore, the actual epidemiological status of SRLVs in wild ruminants is unknown.

Data on the detection of SRLVs proviral DNA in wild ruminants are also limited. In Poland, tests to detect SRLVs in free-living ruminants have never been conducted before. However, as previous studies have shown, CAEV infection was possible in mouflon hybrids after experimental infection with a molecular clone, while natural SRLVs infection has been confirmed in Alpine and Rocky Mountain goats [11, 19–21]. In the case of Rocky Mountain goats, immunohistochemical staining of tissues from the lungs, spinal cord and joints confirmed CAEV infection [11], while SRLVs proviral DNA was successfully obtained from ibexes living in the French Alps. The sequences obtained from ibexes were more closely related to CAEV than to MVV but were quite distinct from sequence of the reference Cork strain [19, 20]. In addition, the sequences obtained were more closely related to those obtained from infected goats that had close contact with these ibexes, which undoubtedly supports the thesis of interspecies SRLVs infection of wild ruminants and adaptation to this new host under natural conditions [19]. In this study, CAEV-like sequences were detected in two red deer, and these sequences were also quite different from those of the Cork strain, with a genetic distance of 12.0%. Although group A (MVV-like) SRLV strains predominate in Polish sheep and goats, only CAEV-like strains were detected. This may suggest that CAEV-like strains are more prone to cross the species barrier and cause persistent infection in wild ruminants. Unfortunately, only *LTR-gag* sequences were obtained from red deer. Despite the fact that the SRLV *gag* and *pol* genes are relatively conserved, we were unable to amplify these fragments. This may suggest that host adaptation after interspecies transmission is related to changes in viral gene sequences that can counteract the restriction factors of the new host [36].

We were able to detect SRLVs proviral DNA in only two red deer, representing 0.64% of all the tested animals, which indicates that interspecies transmission from sheep/goats to red deer occurs at a low level. PCR may fail to detect the virus when the viral load is below the test threshold. However, the nested real-time PCR method used in this work proved to be highly sensitive, as it was able to detect 5 genome copies of SRLV per reaction. The difficulty in isolating retroviral genetic material from wild ruminants was also observed by Materniak et al., who were able to amplify proviral DNA of Foamy virus (FV) in only one out of 269 tested samples (0.37%), although the presence of specific antibodies was detected in 30% of bison and 7.5% of the deer sera. The authors successfully obtained *pol* and LTR fragments but failed to amplify the *gag* fragment [37]. PCR is less sensitive than serological tests and depends mainly on the specificity of the designed primers and the heterogeneity of the viral genome. Mutations consistently generated in the SRLV genome complicate the design of effective primers for the molecular detection of SRLVs and lead to amplification failure. One of the most conserved regions of the lentiviral genome is the RNAt^lys^ primer binding site (PBS) and the nucleotide sequence where the single-stranded primer binds to initiate replication [3, 38]. For amplification of the *LTR-gag* fragment used in this study, primers annealed to such regions were used in the first round of amplification. In addition, primers that bind to sequences located between the major splicing donor (MSD) and the *gag* initiation codon were used in the second round of amplification. These sequences are highly conserved in sheep and goat lentiviruses, implying a critical functional role for encapsidation [39, 40]. The high similarity of the sequences obtained from the two deer in this work supports this thesis. Therefore, we speculate that the choice of such primers for PCR enables SRLVs amplification in red deer. HIV studies have also shown that the most conserved part of the HIV genome is located in the 5’ untranslated leader region rather than in one of the open reading frames [41]. For this reason, *LTR-gag* PCR may be more sensitive for SRLVs diagnosis than PCR based on the *gag* and *pol* regions [42]. The low number of detected wildlife carrying SRLV proviral DNA may also result from the natural clearance of SRLVs in heterologous hosts. This phenomenon has been observed by Morin and coworkers, who revealed that experimental infection of newborn calves with CAEV caused productive but not persistent infection. Integration of proviral DNA into the leukocytes and tissues of calves was confirmed, but the infection did not persist longer than 4 months. After this time, calves clear the virus from their bodies [43]. Furthermore, the PCR used in this work was designed to detect only SRLV isolates belonging to groups A and B. Isolates belonging to other groups that may circulate in free-living small ruminants may not be detectable using this method.

It is unknown whether the SRLVs detected in red deer in this study was capable of replication or existed as a latent reservoir of the virus. CAEV has been shown to have broad host tropism in vitro, causing productive infection in the cells of many wild and domestic ruminants [44, 45]. Sanjose et al. showed that deer skin fibroblast cells were susceptible to SRLVs infection. Proviral DNA from red deer cells was detected in cells infected with various SRLV strains, but RNA production was confirmed only for EV-1 strain infection and only 48 h after inoculation. The authors also showed that although SRLV strains were able to enter deer cells, they differed in their ability to penetrate these cells [13]. It is not entirely clear what such differences might be due to. Perhaps they may be due to the use of different receptors, as differences in receptor use have been observed between MVV and CAEV [46].

It is also worth noting that the CAEV-like sequences detected in this study were isolated only from red deer. In a previous study, we noted that although antibodies against SRLVs were detected in both red deer and roe deer, higher reactivity was observed in red deer [22]. This may suggest that red deer are more susceptible to SRLVs infection, but this needs confirmation. For successful interspecies transmission, contact must occur between the source of the virus and a susceptible host. Since there are no farms in Poland where wild ruminants are kept together with sheep and goats, it is speculated that shared pastures may be a risk factor for SRLVs transmission from infected sheep and goats to wild ruminants during the wild grazing season. The fact that positive animals were detected only in the Carpathian Province, where traditional sheep grazing is practiced (April to September) in Poland, may support this assumption. Such SRLVs transmission can occur through direct contact with an infected host or, more likely, through ingestion of contaminated water, feed and excretory products in the environment. Infection via the fecal-oral route is also possible, as small amounts of SRLVs are excreted in feces [10]. In addition, deer live in larger groups than roe deer, suggesting that interspecies transmission of the virus through direct contact is also likely [47). The detection of SRLV DNA in deer at two locations close to each other may indicate that the animals were from the same herd.

In conclusion, the results of this work indicate that red deer can carry SRLV proviral sequences related to CAEV; therefore, red deer may play a role in the epidemiology of SRLVs. To our knowledge, this is the first study describing SRLV sequences from red deer. These sequences may originate from domesticated sheep/goats grazing on the same pastures, which may increase the risk of virus transmission to livestock. Further research is needed to determine whether the adaptation of SRLV to free-living hosts may involve the acquisition of new genetic and biological characteristics that could threaten not only goats and sheep but also new host species.

### Electronic supplementary material

Below is the link to the electronic supplementary material.

Supplementary Material 1

## Data Availability

All novel sequences obtained in this study were deposited in the GenBank database under accession numbers PP054400-PP054401.
