# Supplementary figures and images for: Detection of small ruminant Lentivirus proviral DNA in red deer from Poland
Source: BMC Vet Res. 2024 May 13;20:195. doi: 10.1186/s12917-024-04059-y (PMC11089798; doi:10.1186/s12917-024-04059-y)

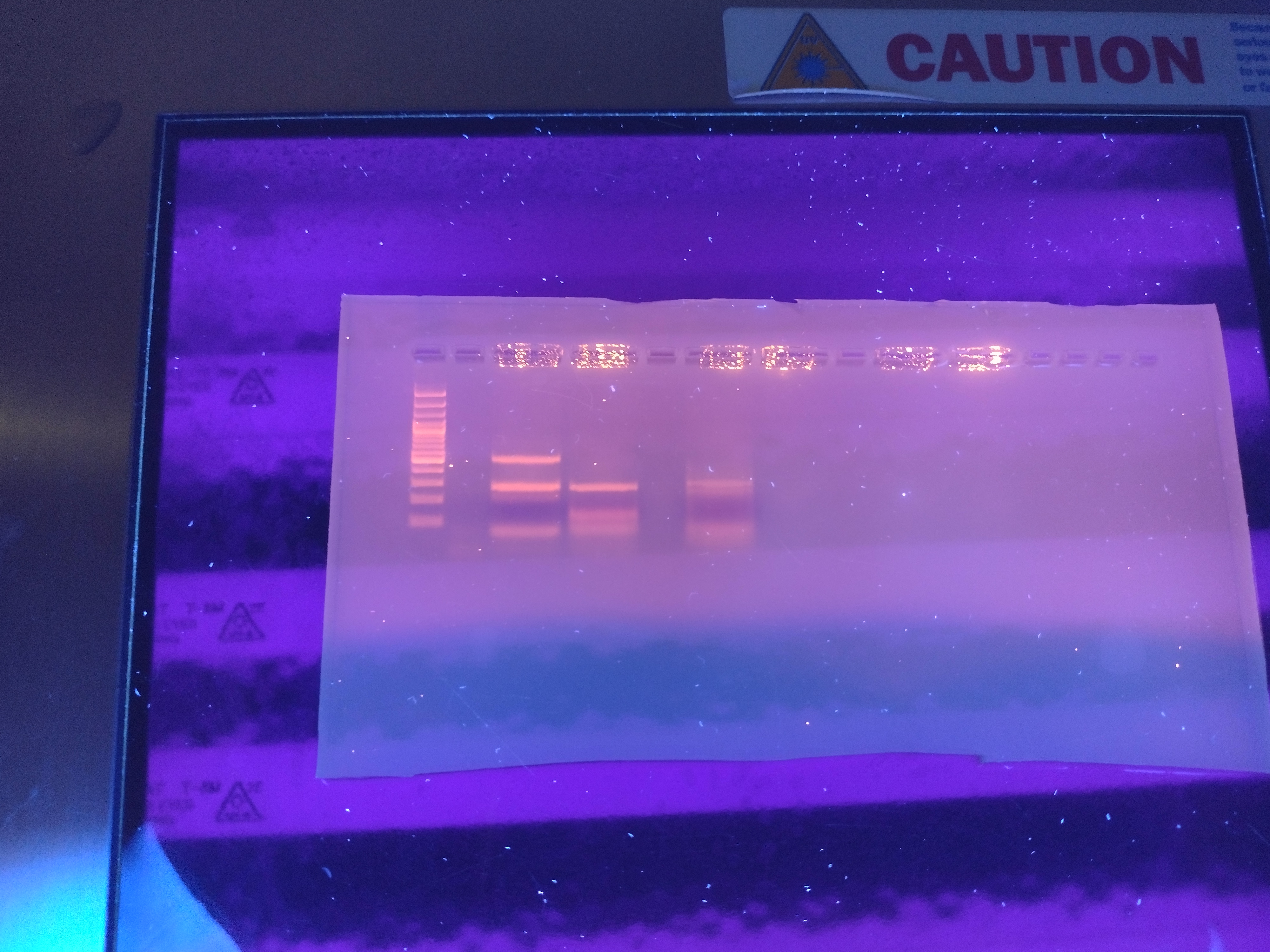

Supplement: Supplementary file 1 — Supplementary Material 1 [file 12917_2024_4059_MOESM1_ESM.jpg]
